# Exploring the Risk and Protective Factors for the Mental Health of Sexual Minority Asian Americans

**DOI:** 10.1093/geroni/igaa057.2127

**Published:** 2020-12-16

**Authors:** Jason Flatt, Rachel Whitmer, Paola Gilsanz

**Affiliations:** 1 University of Nevada, Las Vegas, School of Public Health, Las Vegas, California, United States; 2 University of California Davis, Davis, California, United States; 3 Kaiser Permanente, Oakland, California, United States

## Abstract

This study characterizes the mental health of Asian American older adults (aged 60+) who identify as sexual minorities (SM or lesbian, gay, bisexual) and compare to their non-Asian American and non-SM counterparts. Data were from the Research Program on Genes, Environment and Health (Aged 60+; N=185,478), a representative sample of healthcare members from Northern California. It includes SM (N=447) and heterosexual/non-SM (N=15,772) older adults who identify as Asian American (Chinese, Japanese, Filipino, and South Asian) and non-Asian American SM (N=3,890). Rates of dementia, anxiety, and PTSD were similar for both SM and non-SM Asian Americans. However, older lesbian and gay Asian Americans were more likely to have a depression diagnosis (30% vs. 18%, p=0.002) compared to non-SM. Overall, mental health outcomes were lower for Asian American SM compared to non-Asian American SM. We discuss need for understanding protective factors for mental health and implications for future interventions.

